# Synergistic Activity of Rhamnolipid Biosurfactant and Nanoparticles Synthesized Using Fungal Origin Chitosan Against Phytopathogens

**DOI:** 10.3389/fbioe.2022.917105

**Published:** 2022-08-09

**Authors:** Bhoomika M. Karamchandani, Priya A. Maurya, Sunil G. Dalvi, Samadhan Waghmode, Deepansh Sharma, Pattanathu K. S. M. Rahman, Vandana Ghormade, Surekha K. Satpute

**Affiliations:** ^1^ Department of Microbiology, Savitribai Phule Pune University, Pune, India; ^2^ Tissue Culture Section, Vasantdada Sugar Institute, Pune, India; ^3^ Department of Microbiology, Elphinstone College, Mumbai, India; ^4^ Amity Institute of Microbial Technology, Amity University Rajasthan, Jaipur, India; ^5^ TeeGene and TARA Biologics, Life Science Accelerator, Liverpool School of Tropical Medicine, Liverpool, United Kingdom; ^6^ Centre for Natural Products Discovery, School of Pharmacy and Biomolecular Sciences, Liverpool John Moores University, Liverpool, United Kingdom; ^7^ Nanobiosciences Group, Agharkar Research Institute, Pune, India

**Keywords:** agriculture, biosurfactant, *Cunninghamella*, chitosan, chitosan nanoparticles, phytopathogens

## Abstract

Phytopathogens pose severe implications in the quantity and quality of food production by instigating several diseases. Biocontrol strategies comprising the application of biomaterials have offered endless opportunities for sustainable agriculture. We explored multifarious potentials of rhamnolipid-BS (RH-BS: commercial), fungal chitosan (FCH), and FCH-derived nanoparticles (FCHNPs). The high-quality FCH was extracted from *Cunninghamella echinulata* NCIM 691 followed by the synthesis of FCHNPs. Both, FCH and FCHNPs were characterized by UV-visible spectroscopy, DLS, zeta potential, FTIR, SEM, and Nanoparticle Tracking Analysis (NTA). The commercial chitosan (CH) and synthesized chitosan nanoparticles (CHNPs) were used along with test compounds (FCH and FCHNPs). SEM analysis revealed the spherical shape of the nanomaterials (CHNPs and FCHNPs). NTA provided high-resolution visual validation of particle size distribution for CHNPs (256.33 ± 18.80 nm) and FCHNPs (144.33 ± 10.20 nm). The antibacterial and antifungal assays conducted for RH-BS, FCH, and FCHNPs were supportive to propose their efficacies against phytopathogens. The lower MIC of RH-BS (256 μg/ml) was observed than that of FCH and FCHNPs (>1,024 μg/ml) against *Xanthomonas campestris* NCIM 5028, whereas a combination study of RH-BS with FCHNPs showed a reduction in MIC up to 128 and 4 μg/ml, respectively, indicating their synergistic activity. The other combination of RH-BS with FCH resulted in an additive effect reducing MIC up to 128 and 256 μg/ml, respectively. Microdilution plate assay conducted for three test compounds demonstrated inhibition of fungi, FI: *Fusarium moniliforme* ITCC 191, FII: *Fusarium moniliforme* ITCC 4432, and FIII: *Fusarium graminearum* ITCC 5334 (at 0.015% and 0.020% concentration). Furthermore, potency of test compounds performed through the in vitro model (poisoned food technique) displayed dose-dependent (0.005%, 0.010%, 0.015%, and 0.020% w/v) antifungal activity. Moreover, RH-BS and FCHNPs inhibited spore germination (61–90%) of the same fungi. Our efforts toward utilizing the combination of RH-BS with FCHNPs are significant to develop eco-friendly, low cytotoxic formulations in future.

## 1 Introduction

The agricultural sector faces the demand for enhanced productivity to meet the ever-increasing needs of a growing population. The increased global population has disrupted the balance between production and demand, thereby affecting the availability of food. As a consequence, comprehensive agricultural production must be improved by 70% (Gu et al., 2021). Although the agricultural sector is advancing, it is still challenging to satisfy the demands of the starving population across the globe. Crop productivity is affected by a number of factors, including environmental changes, extreme weather, global warming, and disease outbreaks. Approximately $220 billion is lost annually through the outbreak of diseases caused by several pathogens (bacteria, fungi, and viruses). Population growth and an increase in the food supply have led to the enormous use of agrochemicals by farmers (Maluin and Hussein, 2020). The current market size of the agrochemical industry is USD 220 billion and expected to become USD 340 billion in 2027 (compound annual growth rate [CAGR] of ∼3%) over the forecast period of 2021–2027 (Global Agrochemicals Market^1^). Nonetheless, excessive chemical use creates serious concerns, as it pollutes water and air and releases greenhouse gases (Pestovsky and Martinez-Antonio, 2017). Subsequently, this scenario generates innumerable sources of hazards for human health and the environment. In order to overcome these numerous challenges, an eco-friendly sustainable approach is obligatory for the agriculture sector.

The green approach involves the use of multifunctional biomolecules like biosurfactants (BSs), chitosan (CH), and chitosan-derived nanoparticles (CHNPs) mainly due to their biocompatible nature. The BSs (Sachdev and Cameotra, 2013), CH, and CHNPs (Malerba and Cerana, 2018; Mazzotta et al., 2022) have been explored for plant growth promotion and as biocontrol agents. BSs have been investigated for enhancing nutrient availability to plants, encouraging symbiotic associations, and improving soil health through the bioremediation of heavy metals, hydrocarbons, and other contaminants (Kumar et al., 2021). The BSs are nontoxic, biodegradable, and eco-friendly molecules that exhibit exceptional antimicrobial potential. Consequently, the wide applicability in improved crop and food safety certifies the BS-based biocontrol formulation in sustainable crop management strategies (Sharma, 2021). The antimicrobial potential of BSs can be broadened with other biomaterials like CH and CHNPs for innovative applications. Chemically, CH is the deacetylated derivative of chitin consisting of β-(1,4)-2-acetamido-2-deoxy-d-glucose and β-(1,4)-2-amino-2-deoxy-d-glucose units. The CH biopolymers originated from crustacean and fungal sources (FCH) are commercially valued product. Fungi of the Zygomycetes group efficiently convert chitin to CH by an enzyme—chitin deacetylase. FCH produced by Zygomycetes fungi is vastly recognized due to their high degree of deacetylation (DDA) and solubility. Additionally, the homogenous polymer length of FCH proposes abundant advantages over crustacean CH (Ghormade et al., 2017). CH and FCH are being explored continuously due to their broad-spectrum antimicrobial potential (Karamchandani et al., 2022). The matrix of these biopolymers functions as a shielding reservoir for the release of active ingredients by protecting them from the surrounding environment and controlling the release of agrochemicals (Cota-Arriola et al., 2013). CH biomolecule is also an active plant elicitor with massive antimicrobial potential. The well-appreciated biopolymer enables the delivery of agrochemicals and prevents postharvest decay (Karamchandani et al., 2022). Nanomaterials due to their unique structure, chemical composition, small particle size, and solubility have attracted the scientific fraternity, particularly for the purpose of agrochemicals. It is now more than past three decades that NPs have received considerable acceptance due to their exceptional functional attributes (Staroń and Długosz, 2021). Currently, nanotechnology stands as one of the most promising approaches to overcome the shortcomings of conventional agrochemicals and protecting crops successfully. The CH-derived CHNPs have superior antimicrobial action as compared to bulk materials due to their polycationic nature (Hosseinnejad and Jafari, 2016). In 2016, Sathiyabama and Parthasarathy reported that FCH and FCHNPs pose employability for formulation/products due to their adsorption abilities, biocompatibility, biodegradability, nontoxicity, and cost-effectiveness.

The agricultural sector needs to explore nano-empowered solutions to meet the food requirements in the global framework (Pestovsky and Martínez-Antonio, 2017). Our recent overview on CH and its derivatives facilitated in the realization of their promising role in averting fungal diseases of crops (Karamchandani et al., 2022). The protocols and nanoagrochemicals are to be well thought out for multifarious molecules discretely and/or in combination (Kah and Kookana, 2020). The combination of BS with FCH and/or FCHNPs undoubtedly offers superlative applications in agriculture. After a thorough literature survey, we realized that there is no solitary report on RH-BS in combination with FCHNPs synthesized from FCH of *C. echinulata*. In view of this background, we explore the multifunctionalities of commercially available RH-BS and synthesized FCHNPs against phytopathogens associated with citrus, wheat, and sugarcane, etc. First, FCH was extracted from *C. echinulata* NCIM 691 and used for the synthesis of FCHNPs. We have also characterized FCH and FCHNPs meticulously to assure their authenticity. The antibacterial and antifungal activities of FCH and FCHNPs were investigated individually and in combination with RH-BS. Inhibition of fungal phytopathogens was also demonstrated through poisoned food technique and spore germination assays to propose innovative applications of RH-BS and FCHNPs in agriculture.

## 2 Materials and Methods

### 2.1 Microorganisms: Fungal Culture and Phytopathogens

All the microbial cultures used in the study were procured from various repositories in India. The fungus *Cunninghamella echinulata* NCIM 691 and the bacterium *Xanthomonas campestris* NCIM 5028 were procured from the National Collection of Industrial Microorganisms (NCIM), Pune, Maharashtra, India. The ecologically heterogeneous and predominantly terrestrial fungus *C. echinulata* NCIM 691 was used for the extraction of FCH from its cell wall. Furthermore, extracted FCH was used to synthesize FCHNPs. The bacterial phytopathogen *X. campestris* NCIM 5028 was used for antibacterial assays. Other three fungal phytopathogens (labeled as FI, FII, and FIII), namely, FI: *Fusarium moniliforme* Sheldon ITCC 191, FII: *Fusarium moniliforme* Sheldon ITCC 4432, and FIII: *Fusarium graminearum* Schwabe ITCC 5334 were procured from the Indian Type Culture Collection (ITCC), Delhi, India, and used for antifungal assays (poisoned food technique and inhibition of spore germination). All the cultures were grown and maintained in media and growth conditions as per supplier’s instructions.

### 2.2 Materials

The rhamnolipid-BS (RH-BS) was purchased from AGAE Technologies, United States. The RH-BS is composed of a mixture of mono- and di-rhamnolipid having a purity of 90%. Sodium tripolyphosphate (TPP) and commercial chitosan: CH (molecular weight = 50–190 kDa, degree of deacetylation 75%–85%) were purchased from Sigma-Aldrich (Merck KGaA, Darmstadt, Germany). The other materials used were of analytical grade. Pure quality and dehydrated media like malt extract glucose yeast extract peptone (MGYP), potato dextrose broth/agar (PDB/PDA), Mueller–Hinton broth/agar (MHB/MHA), and Luria–Bertani broth (LB) were purchased from HiMedia (Mumbai, India).

### 2.3 Extraction of Fungal Chitosan From *C. echinulata*

The fungal culture *C. echinulata* NCIM 691 was revived by growing in potato dextrose broth (PDB). Further conditions were set to extract FCH from the fungal mycelia using the alkali-insoluble method (Pochanavanich and Suntornsuk, 2002). First, *C. echinulata* NCIM 691 was grown on potato dextrose agar (PDA) medium for up to 7 days. Furthermore, the culture was scraped gently in phosphate buffer saline (PBS) to prepare the spore suspension (1.0 × 10^8^ spores/ml). The spore suspension was inoculated in 250 ml Erlenmeyer flasks containing 100 ml of PDB and were incubated at 30°C for 16 h on a rotatory shaker (180 rpm). Next day, about 7.5% (v/v) of 16 h old inoculum was added aseptically to the 500 ml Erlenmeyer flasks with 200 ml of the sterile PDB. The flask was kept on a rotary shaker (180 rpm) at 29 ± 1°C for 12 days. The fungal biomass was initially separated from the media by filtration (Whatman filter paper, Grade No. 1—size 110 mm) and dried until the homogenous weight was obtained. The dry weights of the biomasses were noted, and then alkaline treatment was carried out using 1 N NaOH (1:30 w/v). This process helped in the removal of proteins and glucans from the alkali-treated supernatant. Subsequently, the solution was autoclaved at 121°C for 15 min. The alkali-insoluble material was centrifuged at 10,000 rpm for 15 min (Kubota AG-5006A, Tokyo, Japan). Post centrifugation, distilled water was added accordingly to reduce the pH up to 7.0. The obtained substance was dried further in a hot air oven at 60°C until a constant weight was achieved. This step was followed by the treatment of acetic acid (1% v/v) to the insoluble mass and was kept in a hot water bath (95°C) for 5 h. This imperative step dissolved the FCH components and was followed by centrifugation at 10,000 rpm for 20 min. The supernatant was collected and treated with 2 N NaOH to bring the pH up to 10.0. This was followed by another round of centrifugation at 10,000 rpm for 20 min, and the pellet was collected. This extracted FCH was washed multiple times with distilled water followed by 95% ethanol (1:20) and further with acetone (1:20). The extract was kept for drying in a hot air oven at 60°C until the homogenized weight could be obtained. This extract of FCH was ready for the synthesis of FCHNPs and stored at 4°C until further use.

#### 2.3.1 Synthesis of Chitosan Nanoparticles From Fungal Chitosan of *C. echinulata*

The FCH extracted from the fungus *C. echinulata* NCIM 691 was employed to synthesize FCHNPs through the ionic gelation method as described by de Carvalho et al. (2019). Briefly, FCH was dissolved in 2 mg/ml acetic acid (1% v/v), and pH was adjusted to 5.0. The solution of sodium tripolyphosphate (TPP) was prepared in distilled water at 0.1% (w/v). About 5 ml of TPP solution was added to 15 ml of the FCH. This quantification of solutions depicts the ratio of 3:1 for CH:TPP (de Pinho Neves et al., 2014). The TPP solution was filtered through a 0.2 µm membrane filter (Pall Life Sciences, USA) and was added to FCH solution dropwise under stirring conditions using a magnetic stirrer at 700–800 rpm (room temperature) to obtain a clear solution (de Carvalho et al., 2019). After the complete addition of TPP, the solution was left under stirring for another 30 min. The positively charged amino acid group of FCH interacts with the negative charge of TPP resulting in the ionotropic gelation reaction. The ionic gelation method of FCH synthesis initiated with meshwork formation upon TPP addition and enabled the synthesis of FCHNPs. Afterward, the solution was centrifuged at 10,000 rpm for 20 min (Khoerunnisa et al., 2021). The FCHNPs were obtained in the form of precipitate and subsequently washed twice with distilled water to remove the unreacted substances. The purified FCHNPs were then freeze-dried and stored at 4°C until further use. A similar protocol was used to synthesize CHNPs from commercial CH (Sigma-Aldrich, Merck KGaA, Darmstadt, Germany). The physicochemical characterization of FCH and FCHNPs was carried out using various analytical techniques as mentioned in the following sections. We had included commercial CH and its synthesized CHNPs as references to analyze and compare the data for FCH and FCHNPs, respectively.

#### 2.3.2 Physicochemical Characterization of Fungal Chitosan and Chitosan Nanoparticles

The FCH and FCHNPs were characterized using the following analytical techniques: 1. UV-visible spectroscopy, 2. Dynamic Light Scattering (DLS), 3. Zeta potential, 4. Fourier Transform Infra-Red Spectroscopy (FTIR), 5. Scanning Electron Microscopy (SEM), and 6. Nanoparticle Tracking Analysis (NTA). All the samples were prepared and used as per the requirements of the corresponding technique. Appropriate reference samples were also included in the particular analytical techniques.

### 2.4 UV-Visible Spectroscopy

The UV-visible spectrophotometry (UV–Vis) is a routinely used technique for the quantitative determination of analytes, chemicals, and biological macromolecules. The UV-visible spectra of FCH and FCHNPs were recorded using a Jasco V-770 Spectrophotometer (JASCO Deutschland GmbH, Pfungstadt, Germany) to confirm the absorption spectra in the range of 200–800 nm (Agarwal et al., 2018; Khoerunnisa et al., 2021). This analytical tool was employed to examine FCH and FCHNPs samples which were also compared with CH and CHNPs.

### 2.5 Dynamic Light Scattering and Zeta Potential

Dynamic light scattering (DLS) is the most widely used technique to determine the hydrodynamic diameter of NPs and afford evidence on the aggregation state of NPs in solution. The technique determines the average particle size on a high-performance particle Zetasizer HPPS-5001 (Malvern, UK). The CH, CHNPs, FCH, and FCHNPs were analyzed in triplicate at 25°C at a scattering angle of 90°. Pure distilled water was used as a reference dispersing medium. All the samples were dissolved in 0.5% acetic acid followed by sonication for 15 min in an ultrasonicator (QSonica Sonicator, USA). Samples were then rinsed with distilled water followed by filtration using a syringe filter of 0.2 µm pore size (Pall Life Sciences, USA), and the average particle size was analyzed in triplicates (Ghadi et al., 2014).

The zeta potential of all the samples was measured using a Zetasizer Nano (Malvern, UK) to manifest the effective electric charge on the surface of NPs and quantify the charges. The property of zeta potential depends on the surface charge which is extremely significant for the stability of NPs in suspension. The zeta potential is also a key feature during the adsorption of NPs at the initial stage onto the membrane of any cell. After the adsorption of NPs on the cell surface, the rate of their uptake is dependent on the particle size. Thus, nanosize and zeta potential contribute meaningfully to the toxicity of NPs. The electrophoretic mobility of the samples assisted in depicting their zeta potential.

### 2.6 Fourier Transform Infra-Red Spectroscopy

Fourier transform infrared (FTIR) spectroscopy is a sensitive technique that recognizes the presence of functional groups. The technique is fairly suitable to identify and classify vital biomolecules that can be employed for diverse applications. The completely dried samples of FCH and FCHNPs were examined. The structural analysis was carried out on a spectrophotometer (Alpha II, Bruker, UK) in a wavenumber range of 400–4,000 cm^−1^. FTIR spectra of all biomaterials were attained by using 1 mg of sample. The spectrum was then recorded and compared with the commercial CH and CHNPs (Pawlak and Mucha, 2003; Divya et al., 2017). Confirmation of scans was carried out to achieve a decent signal/noise ratio.

### 2.7 Scanning Electron Microscopy

The advancements in scanning electron microscopy (SEM) empower the high-resolution imaging of biomaterials. Both the nanoparticles (CHNPs and FCHNPs) were air-dried and were mounted on silicon wafers to be used to divulge their size, texture, and shape. The shape and surface morphology were determined using the SEM setup of FEI Nova NanoSEM 450, USA. The samples were dissolved in distilled water and placed on silica wafers (Sigma-Aldrich, USA), which were allowed to dry completely. Both the samples were then coated with gold and observed at an acceleration voltage of 10.0 kV with the highest magnification of ×50,000 (Anitha et al., 2009).

### 2.8 Nanoparticle Size Analysis

The size of the NPs was also confirmed through nanoparticle tracking analysis (NTA). This technique employs light scattering as well as Brownian motion to estimate NP size distribution of samples. The size analysis of the NPs was carried out through nanotracking analysis (NTA 2.3, UK) (Rahi et al., 2022). The sample was loaded in liquid suspension into a chamber with a laser beam for sample illumination scattering the laser light. The NTA technique needs a sample in low volumes without any sophisticated protocols for the sample preparation. The ×20 microscope objective was used to observe the images which were captured using a digital camera. The camera captures a video of the particles moving under Brownian motion. Several particles individually and simultaneously (particle-by-particle) are analyzed by NTA software. Hence, the NanoSight instrument provided NP size in high resolution, concentration, and aggregation measurements (count based) of CHNPs and FCHNPs. Concurrently, fluorescence mode provided specific results for suitably labeled particles. The real-time monitoring process helped to scrutinize the subtle changes in the particle population characterization.

### 2.9 Application of Rhamnolipid Biosurfactant, Fungal Chitosan, and Synthesized Chitosan Nanoparticles in Controlling the Selected Phytopathogens

This section dealt with evaluating the efficacies of the test compounds—RH-BS (commercial), FCH (extracted), and FCHNPs (synthesized) against selected phytopathogens. The commercial CH and its synthesized CHNPs (by us in the laboratory) were tested against four phytopathogens. *X. campestris* NCIM 5028 (causative agent of black rot and bacterial wilt, etc.) and three fungi: FI (causative agent of the stalk rot, ear rot, and kernel rot of corn), FII (causative agent of the stalk rot, ear rot, and kernel rot of corn), and FIII (causative agent of the Fusarium head blight on wheat, barley, and other grains) were included in the assays. The following experiments from 1 to 3 were performed against the bacterium *X. campestris* NCIM 5028. Experiment numbers 4 to 6 were performed against three fungal pathogens (FI, FII, and FIII).

1. Determining the minimum inhibitory concentration of the test compounds against *X. campestris* using microdilution assay.
2. Determining the synergistic activity of the test compounds against *X. campestris* using checkerboard assay.
3. Evidencing antibacterial effect of the test compounds against *X. campestris* using SEM.
4. Determining the antifungal activity of the test compounds against three fungal phytopathogens using microdilution assay.
5. Determining the efficacy of the test compound dosages against three fungal phytopathogens through the poisoned food technique.
6. Determining the efficacy of the test compounds in inhibiting the spore germination of three fungal phytopathogens.

#### 2.9.1 Determining Minimum Inhibitory Concentration of the Test Compounds Against *X. campestris* Using Microdilution Assay

The minimum inhibitory concentration (MIC) of three test compounds, viz., RH-BS, FCH, and FCHNPs, was determined against the bacterial pathogen *X. campestris* NCIM 5028. This culture was grown in LB at 30°C, and after 48 h of incubation, the bacterial suspension was prepared in MHB. The inoculum for the microdilution assay was prepared by adjusting the optical density (OD_620_) value equivalent to 10^8^ CFU/ml (determined from a calibration curve). The MIC for all the test compounds was performed by broth dilution method in separate 96-well microtiter plates (Zgoda and Porter, 2001). All three test materials were dissolved in MHB at twice the concentration of the final test compounds, with pH adjusted to 7.0. Furthermore, 100 µl of MHB was dispensed in each well of a microtiter plate (Column 3–12) containing MHB only. Column 1 contained 100 µl of dilute culture inoculum (OD adjusted) (as mentioned previously), and Column 2 contained 100 µl of the medium broth (as a negative control to monitor sterility). A multichannel pipette was then used to transfer and mix the test compounds from Columns 12 (200 µl) to 3, resulting finally a volume of 100 µl per well. The concentrations between 2 and 1,024 µg/ml were achieved for all the three test compounds through double dilutions serially from Columns 12 to 3. The absorbance of samples was measured at 620 nm at 0 and 48 h. After completing the incubation period, the MIC was identified from the well showing no visible growth in the presence of the test compound. Thus, the lowest concentration of the test compound inhibiting the visible growth of the organisms was documented as MIC. Experiments were performed in triplicate, and the mean values of three independent biological replicates were considered.

#### 2.9.2 Determining the Synergistic Activity of the Test Compounds Against *X. campestris* Using Checkerboard Assay

The checkerboard assays were performed to investigate the synergistic activity of all three test compounds (RH-BS, FCH, and FCHNPs) against *X. campestris* NCIM 5028 by following the protocols as described by Okoliegbe et al. (2021) and Mirzaei-Najafgholi et al. (2017). Briefly, 24 h old culture of *X. campestris* NCIM 5028 was inoculated into MHB and incubated at 30°C until the exponential growth phase. Around 100 µl of each dilution of all the test compounds (2 × MIC, 1 × MIC, 1/2, 1/4, 1/8, 1/16, 1/32, and 1/64 MIC) were dispensed to each row. Furthermore, 100 µl of the second compound was added to the same or each row of the wells in the direction perpendicular to the previous compound in different dilutions in a 96-well plate. A two-fold diluted freshly prepared solution of the test compound (at different concentrations) was dispensed in a checkerboard array and inoculated with 10^8^ CFU/ml of the bacterial suspension. The well without any test compound was considered the positive control. The well with the growth medium without inoculum and the test compound was used as the negative control. After incubation at 30°C, plates were examined for visual turbidity using the microplate reader (Spectra Max M2, USA). A fractional inhibitory concentration index (FICI) of both combinations (RH-BS + FCH and RH-BS + FCHNPs) is calculated (Konaté et al., 2012) using the following formula:

$$\begin{array}{l} \mathrm{FICI=FIC}\;A\;\mathrm{+FIC}\;B,\\ FICI=\;\frac{\mathrm{MIC of Test Compound A combined}}{\mathrm{MIC of Test Compound A alone}}+\frac{\mathrm{MIC of Test Compound B combined}}{\mathrm{MIC of Test Compound B alone}}. \end{array}$$

Interaction between combinations of two test compounds is evaluated if the FIC index was: ≤0.5, synergistic; 0.5 < FICI <1, additive; 1 < FICI ≤4, indifferent and FICI >4, antagonistic.

#### 2.9.3 Evidencing Antibacterial Effect of the Test Compounds Against *X. campestris* using SEM

The SEM was performed to evident the effect of all the three test compounds (RH-BS, FCH, and FCHNPs) on *X. campestris* NCIM 5028. The protocol for the same was adopted from Fischer et al. (2012). Cells of *X. campestris* were grown for 24 h at 30°C in MHB. About 10^8^ CFU/ml cells were treated at their respective MIC of the test compound for 24 h at 30°C. The cells without the treatment of the test compounds were encompassed as a negative control. This enabled us to distinguish the morphology of the *X. campestris* NCIM 5028 cells in the presence (test) and absence (control) of the test compounds. The treated cells were washed with PBS by centrifuging at 10,000 rpm for 15 min. The cell pellet obtained after centrifugation was treated overnight with 2.5% glutaraldehyde (Sigma-Aldrich, USA) at 4°C. The next day cells were washed (to remove glutaraldehyde) with PBS by centrifugation at 10,000 rpm for 15 min followed by the ethanol treatment. The first dehydration step was achieved by taking water:ethanol in the ratio of 90:10 and incubating for 15 min, which were then centrifuged at 10,000 rpm at 4°C. The same steps were repeated several times with the different proportions of water and ethanol in the ratio of 80:20, 60:40, 40:60, 20:80, and 10:90, and finally with 100% ethanol. The treated cells were loaded on silica wafers (Sigma-Aldrich, USA), and furthermore ethanol was evaporated completely overnight. The SEM images of the dried sample were analyzed using Nova Nano SEM 450 instrumentation facility (USA) under ×50,000 magnification.

#### 2.9.4 Determining the Antifungal Activity of the Test Compounds Against Three Fungal Phytopathogens Using Microdilution Assay

The MIC of all three test compounds (RH-BS, FCH, and FCHNPs) was evaluated against three selected fungi FI, FII, and FIII. The different concentrations (0.005%, 0.010%, 0.015%, and 0.020% w/v) of the test compounds were considered to determine their antifungal activity. In the microtiter plate, we added the test compound (20 µl) with PDB (120 µl) and 10 µl homogenous fungal suspension (1.0 × 10^8^ spores/ml). The commercial fungicide carbendazim (CBD) (Sigma-Aldrich, USA) of similar concentrations was used as a positive control. The CBD is a commercial, broad-spectrum fungicide popular for controlling several dreaded fungi associated with cereals and fruits like citrus, pineapples, strawberries, and bananas. First, plates were placed in a refrigerator (at 4°C for 1 h) for initial prediffusion and to achieve homogeneous diffusion of the test biomaterials in the surrounding medium in the agar plate. Furthermore, plates were removed from the refrigerator and placed in an incubator (29 ± 1°C for 5 days) for optimum growth. After 5 days of completing the incubation period at 29 ± 1°C, each well was observed with naked eyes under the bright light for fungal mycelial growth (Elshikh et al., 2016; Chopra et al., 2020).

#### 2.9.5 Determining the Efficacy of the Test Compound Dosages Against Three Fungal Phytopathogens Through the Poison Food Technique.

This technique was carried out to confirm the efficacy of all the test compounds (RH-BS, FCH, and FCHNPs) (Saharan et al., 2013) against three *Fusarium* species (FI, FII, and FIII). Different doses (0.005%, 0.010%, 0.015%, and 0.020% w/v) of all test compounds were mixed with potato dextrose broth and poured in Petri dishes (90 mm × 15 mm, HiMedia, Mumbai, India) individually. The mycelial bit of test fungal phytopathogens from the peripheral end of uniform size (diameter, 5.0 mm) were taken from 7-day-old culture and placed in the center of the Petri dishes impregnated with the test compounds individually. These Petri plates were then incubated at 29 ± 1°C for 7 days for the observation of radial mycelial growth. The inoculated plates were compared with the positive control (with commercial fungicide CBD) and negative control (without test compounds) to calculate the percentage inhibition rate of the mycelia for the respective phytopathogen.

$$\%\;\mathrm{Inhibition rate}\;=\;\frac{M_{c}-M_{t}}{M_{c}}\;\times \;100,$$

where M_c_ is the mycelial growth in the control plate and M_t_ is the mycelial growth in treatment.

#### 2.9.6 Determining the Efficacy of the Test Compounds in Inhibiting the Spore Germination of Three Fungal Phytopathogens

All three test compounds were examined to confirm whether they affect the spore germination of fungal phytopathogens and subsequently contribute to antifungal activities. Various concentrations (0.005%, 0.010%, 0.015%, and 0.020% w/v) of the test compounds were included in the assay (Paul et al., 1993). A suspension of 1.0 × 10^3^ spores/ml of all the fungi was prepared aseptically from a 7-day-old pure culture. The 1:1 ratio of spore suspension and the test compounds (50 μl each) were mixed in microtiter plate in 10 replicates. The plates were incubated at 29 ± 1°C for 24 h followed by microscopic observation using glass slide. The percent inhibition rate was determined by counting the number of spores germinated as compared to control using the bright-field microscope (Zeiss, Germany). The following formula described by Saharan et al. (2013) is used to calculate the inhibition percentage:

$$\%\;\mathrm{Inhibition rate}\;=\;\frac{G_{c}-G_{t}}{G_{c}}\;\times \;100,$$

where G_c_ is the germination in control and G_t_ is the germination in treatment.

### 2.10 Statistical Analysis

All the experiments were performed in triplicate, and the results were arranged as the mean ± standard deviation (SD). Data were then analyzed using ANOVA. The repeated measurements and differences were considered to be significant at a level of *p* < 0.05.

## 3 Results

### 3.1 Biomass Production and Extraction of Chitosan From *C. echinulata*

**FIGURE 1:**
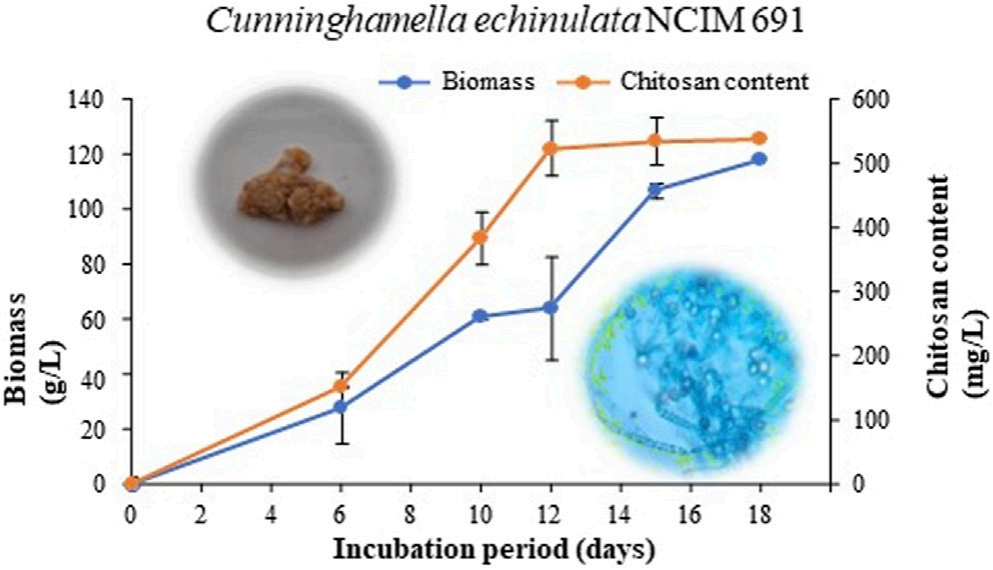
Biomass and chitosan content of *C. echinulata* NCIM 691 grown in potato dextrose broth.

Submerged fermentation carried out by inoculating *C. echinulata* NCIM 691 into a PDB medium (a nutritionally rich) encouraged satisfactory growth of the fungal biomass. The growth curve of *C. echinulata* demonstrating the production of biomass and FCH was carried out successfully for up to 18 days (Figure 1). The lag and exponential phases were seen between 0 and 6 days and 6 and 12 days, respectively. After 12 days of incubation, the fungus exhibited a deceleration phase. The optimal harvesting of FCH was achieved efficaciously on the 12th day of the incubation period. Physicochemical parameters encompassing acidic pH (5.5), aeration (180 rpm), and temperature (30°C) supported a luxurious growth of *C. echinulata* NCIM 691 with a biomass of 64.2 g/L. The maximum yield of FCH found was 523 mg/L under the same cultivation parameters after the 12th day of incubation (Figure 1).

### 3.2 Physicochemical Characterization of Fungal Chitosan and Chitosan Nanoparticles

We have characterized FCH and FCHNPs through several analytical techniques. Commercial CH and its synthesized CHNPs included in the study facilitated confirmation of both test compounds under investigation. Each of the techniques being sensitive could authenticate and confirmed the presence of FCH and FCHNPs in all the test samples. The results for each of the analytical techniques are given in the following sections.

### 3.3 UV-Visible Spectroscopy

This technique displayed unique optical properties of FCH and FCHNPs that are sensitive to shape, size, and concentration. Additionally, the information about the agglomeration state and refractive index near the NP surface enabled the UV-Vis technique as a valued tool for detecting and characterizing the nanomaterial under investigation. The commercial CH and CHNPs depicted absorption at 300 and 290 nm, respectively. The UV-visible spectroscopic graph showed higher intensity absorption peaks in FCHNPs than FCH at 315 and 300 nm, respectively (refer Supplementary Figure S1). The operative technique facilitated analysis of the functional group, their energy transition, and the bandgap also helped in determining the kind of interaction in the compound. Overall, the analysis has successfully measured the extinction (scatter and absorption) of the light after passing through all the samples.

### 3.4 Dynamic Light Scattering and Zeta Potential

The DLS technique was performed to measure hydrodynamic diameter in the nanometer range. The size of commercial CH and the synthesized CHNPs along with FCH and FCHNPs has been illustrated in Supplementary Table S1. The zeta potential measurements revealed that the cationic FCHNPs (45.6 mV > 24.5 mV) possess more charges than CHNPs.

### 3.5 Fourier Transform Infra-Red Spectroscopy

**FIGURE 2:**
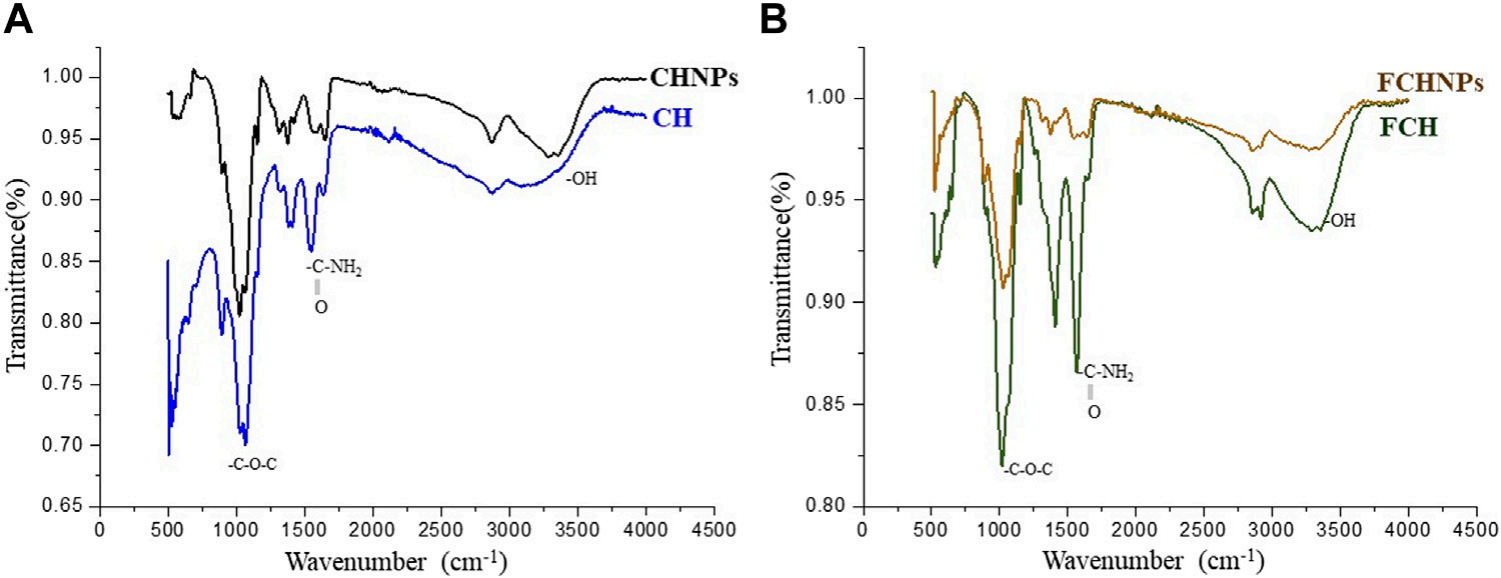
FTIR spectra of **(A)** commercially available chitosan (CH) and synthesized nanoparticles (CHNPs). **(B)** Fungus *C. echinulata*-originated chitosan (FCH) and its synthesized chitosan nanoparticles (FCHNPs).

FTIR absorption spectra of commercial CH, CHNPs, FCH, and FCHNPs are illustrated in Figure 2. For CH, the C-H stretching vibrations were manifested through strong peaks at around 2,869 cm^−1^. A similar characteristic peak (at 2,864 cm^−1^) was also obtained for extracted FCH. The FCHNPs synthesized by us also displayed the distinctive peak at 2,916 cm^−1^. The symmetric stretch of C-O-C was observed at around 1,020–1,057 cm^−1^ in four samples. As seen in both spectra, the strong peaks in the range 3,398–3,346 cm^−1^ correspond to combined peaks of OH and intramolecular hydrogen bonding. The broadness of the peak in this region might be attributed due to the contributions from N-H bond stretches. In FCHNPs, a shift from 3,446 to 3,398 cm^−1^ was observed. A wider peak at 3,461 cm^−1^ was observed for FCH indicating an extensive hydrogen bonding. The characteristic CO-NH_2_ peak of FCH at around 1,557 cm^−1^ was found to be shifted to 1,563 cm^−1^ in the FCHNPs, which could be due to the cross-linking of TPP with the ammonium groups present in FCHNPs. This cross-linking between the TPP and the ammonium groups might be imperative for the inter- and intra-molecular interactions.

### 3.6 Scanning Electron Microscopy

**FIGURE 3:**
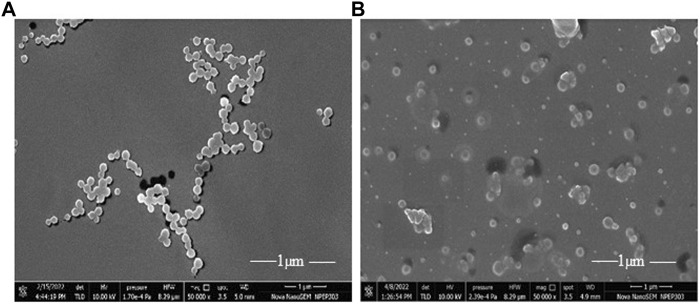
Scanning electron micrograph of synthesized nanoparticles. **(A)** CHNPs from commercial chitosan (CH) and **(B)** FCHNPs from fungal chitosan (FCH) extracted from *C. echinulata* NCIM 691.

The SEM revealed that the particle size of FCHNPs ranges between 70 and 150 nm. The aggregation in FCHNPs occurred due to moisture entrapment. The images captured through the SEM technique showed spherical-shaped FCHNPs (Figure 3). The particle size of CHNPs was found to have a variable range of 200–300 nm.

### 3.7 Nanoparticle Size Analysis

**FIGURE 4:**
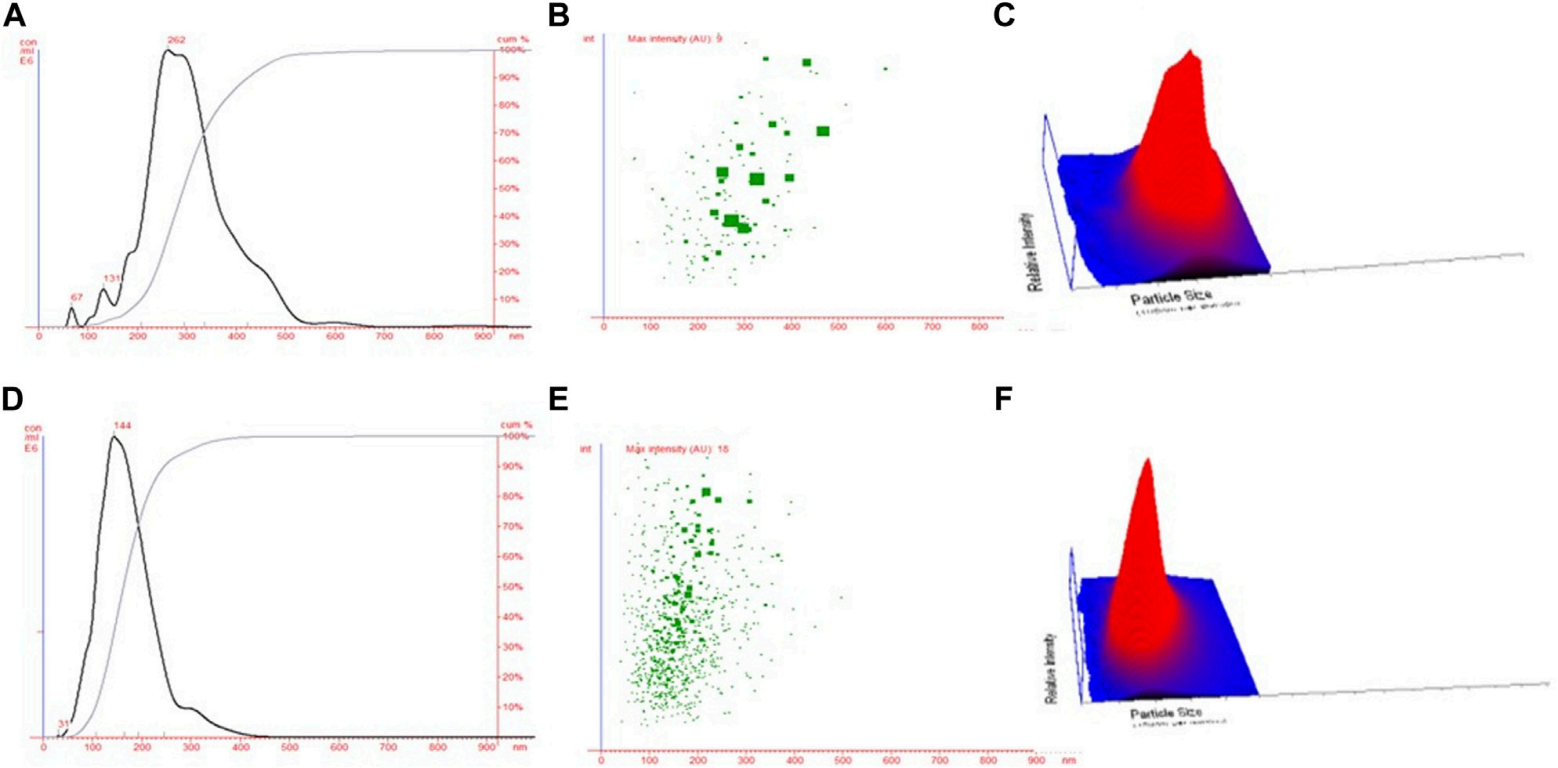
Nanoparticle tracking analysis. **(A)** Particle size versus concentration plot estimating the average size of CHNPs synthesized from commercial CH. Particle size versus relative intensity of CHNPs synthesized using commercial CH in **(B)** scattered and **(C)** 3D plot. **(D)** Particle size versus concentration plot estimating the average size of NPs synthesized from fungal CH extracted from *C. echinulata* NCIM 691. Particle size versus relative intensity of FCHNPs synthesized using fungal CH in **(E)** scattered and **(F)** 3D plot.

The NTA technique assisted in estimating the average size of both NPs (CHNPs and FCHNPs) from the plot of particle size versus concentration as represented in Figure 4. The average size of CHNPs and FCHNPs is represented in Supplementary Table S1. Additionally, the particle size versus relative intensity scattered (Figure 4B) and 3D plot (Figure 4C) confirmed the nature (with respect to homogeneity) of CHNPs synthesized from commercial CH. The size versus relative intensity scattered (Figure 4E) and 3D plot (Figure 4F) demonstrated the superior homogenous nature of FCHNPs as compared with CHNPs. The CHNPs were quite uneven with varied sizes (Figure 4A–C), while a single peak was observed for FCHNPs corresponding to homogeneous particle size (Figure 4D–F). Thus, the NTA methodology manifested the relationship between NP size distribution and concentration successfully.

#### 3.7.1 Microdilution Assay for Determination of MIC of Rhamnolipid Biosurfactant, Fungal Chitosan, and Synthesized Chitosan Nanoparticles

Antibacterial activities of all the test compounds—RH-BS, FCH, and FCHNPs were inspected from MIC against Gram-negative bacterium *X. campestris* NCIM 5028. It was observed that *X. campestris* treated with RH-BS alone had the lowest MIC of 256 μg/ml among the other two test compounds. The MIC for FCH and FCHNPs was found to be > 1,024 μg/ml against the same bacterium. Therefore, the bacterium *X. campestris* NCIM 5028 used in the experiment was resistant to FCH and FCHNPs. It is important to note that the MIC is not actually a single solitary number, nevertheless a range based on the dilution series performed during its determination.

#### 3.7.2 Synergistic Activity of Rhamnolipid Biosurfactant and Synthesized Chitosan Nanoparticles Using Fungal Chitosan Against *X. campestris*

The in vitro synergistic activity of the test compounds in two combinations (RH-BS + FCH and RH-BS + FCHNPs) was carried out against *X. campestris* NCIM 5028. The microdilution checkerboard assay enabled us to determine the synergistic or additive effect of both combinations against the test bacterium. The first combination of RH-BS with FCH could inhibit *X*. *campestris* at sub-MIC levels. The combination between RH-BS and FCH resulted in a reduction in the MIC of RH-BS by one-fold and FCH by more than two folds. In the case of the second combination of RH-BS with FCHNPs, the synergy was comparatively greater since the MIC of RH-BS was reduced to one-fold and of FCHNPs was more than eight folds. Thus, the highest level of synergistic effect was observed between RH-BS and FCHNPs. An FICI of both combinations is denoted in Supplementary Table S2. Overall, we detected a momentous decrease in MICs of RH-BS and FCHNPs suggesting a powerful synergy (FICI 0.503) between this combination. However, the MICs of FCH did not reduce down up to 8 μg/ml against *X. campestris*. Thus, the breakpoint level due to the synergy between RH-BS and FCH could have been observed if there had reduction in MIC of FCH up to 8 μg/ml.

#### 3.7.3 Effect of the Test Compounds on Cell Morphology of *X. campestris*

**FIGURE 5:**
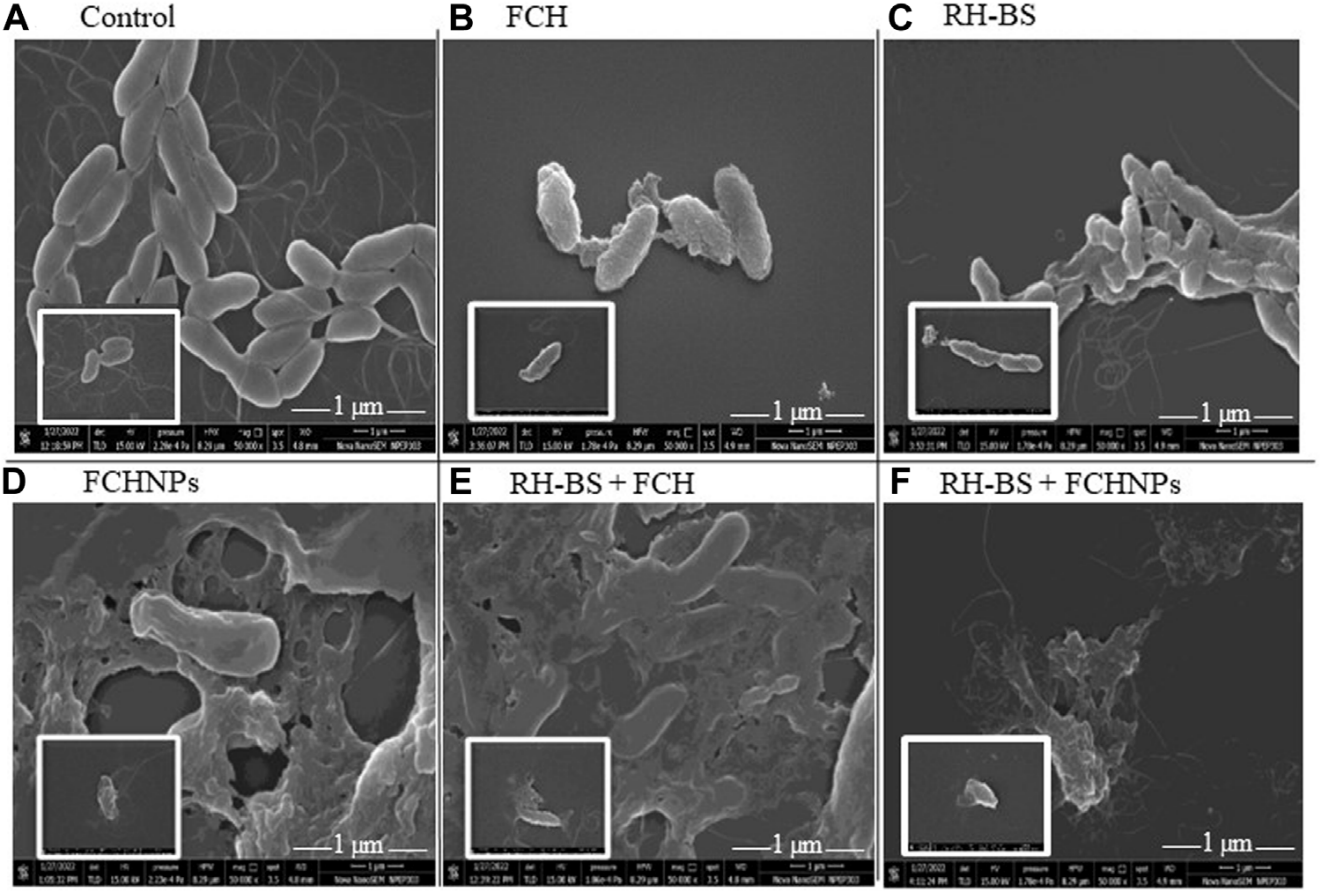
Scanning electron microscopy images (×50,000) demonstrating the effect of three test compounds individually (FCH, RH-BS, and FCHNPs) and in two combinations (RH-BS + FCH and RH-BS + FCHNPs) at MIC (treated for 24 h and incubated at 30°C) on cellular morphology of the bacterial pathogen *Xanthomonas campestris* NCIM 5028. **(A)** Control—intact morphology of the untreated cells. **(B)** Alteration in the cell morphology of pathogen when treated with FCH at MIC (>1,024 μg/ml). **(C)** Alteration in the cell morphology of pathogen when treated with RH-BS at MIC (256 μg/ml). **(D)** Ruptured appearance of the cells of the pathogen when treated with FCHNPs at MIC (>1,024 μg/ml). **(E)** Ruptured appearance of the cells of the pathogen when treated with RH-BS (128 μg/ml) and FCH (256 μg/ml) at MIC. **(F)** Complete distortion of cells of the pathogen due to RH-BS (128 μg/ml) and FCHNPs at lower MIC (4 μg/ml) in comparison to control.

The SEM technique clearly evidenced the effect of test compounds individually (FCH, RH-BS, and FCHNPs) and in both combinations (RH-BS + FCH and RH-BS + FCHNPs) on cellular morphology of *X*. *campestris* NCIM 5028 when treated and incubated on a rotatory shaker for 24 h (at 30°C/180 rpm) at their respective MICs. The control or the bacterial suspension in absence of the test compounds showed very distinct and intact morphology (Figure 5A). The slight alteration in the cell morphology of the pathogen was seen in presence of FCH (MIC: >1,024 μg/ml) (Figure 5B) and RH-BS (MIC: 256 μg/ml) (Figure 5C) during their individual treatment in comparison to control. The cell morphology was well deformed in the presence of FCHNPs when used individually (MICs: 128 μg/ml) (Figure 5D). The SEM manifested the distinctive morphological alteration/destruction of the pathogen after getting treated by the first combination (RH-BS + FCH) (Figure 5E) and a second combination (RH-BS + FCHNPs) (Figure 5F). Consequently, noticeable changes in the morphology of *X. campestris* designated that cell wall, membrane permeability, and viscosity were certainly compromised by synergistic activities of the test compounds.

#### 3.7.4 Antifungal Activity of the Test Compounds Against Selected Phytopathogens

The in vitro assessment of the MIC of all the test compounds was performed against three fungi, viz., FI, FII, and FIII through the microdilution method. The growth of FI and FII was inhibited effectively by RH-BS and FCHNPs at 0.015% concentration. However, the growth of the pathogen FIII was apparent in the presence of all the three test compounds (RH-BS, FCH, and FCHNPs) at 0.015% concentration. In comparison with RH-BS and FCHNPs, the growth of FI and FII was not inhibited effectively by FCH even at the highest concentration (0.020%). These observations were further confirmed by the poisoned food technique as described in the following section. The commercially available fungicide CBD included in the study also inhibited the growth of all three fungal pathogens at the lowest concentration (0.005%). The wells in the absence of all the three test compounds and CBD presented heavy fungal growth.

#### 3.7.5 Poisoned Food Technique

**FIGURE 6:**
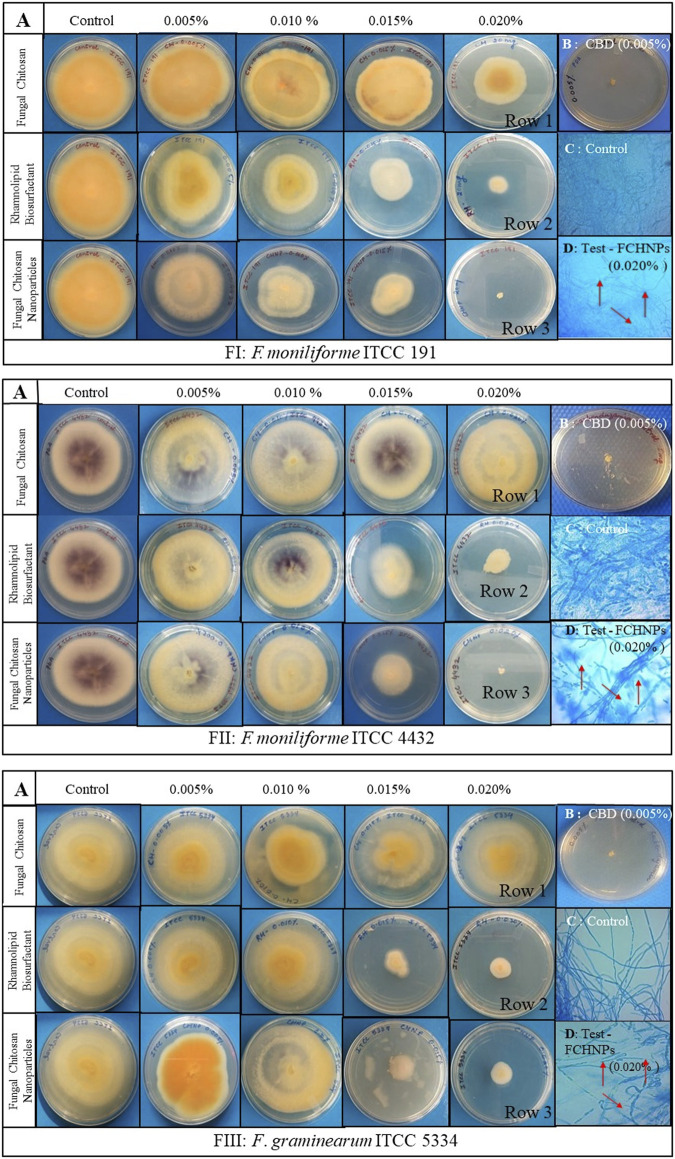
*In vitro* interaction of rhamnolipid biosurfactant (RH-BS), fungal chitosan (FCH), and synthesized chitosan nanoparticles (FCHNPs) against three fungal phytopathogens FI: *F. moniliforme* Sheldon ITCC 191, FII: *F. moniliforme* Sheldon ITCC 4432, and FIII: *F. graminearum* Schwabe ITCC 5334 depicting the morphological alterations. **(A)** Antifungal bioassay demonstrating the effect of the test compounds on mycelial growth of FI, FII, and FIII when treated at different concentrations (0.005%, 0.010%, 0.015%, and 0.020% w/v); **(B)** CBD: 0.005% carbendazim; **(C)** Control: lactophenol cotton blue staining of fungal mycelia without any distortion in absence of the test compounds; **(D)** Test: lactophenol cotton blue staining of the distorted mycelia of fungi when treated with FCHNPs at 0.020% concentration.

A routinely employed in vitro poisoned food technique facilitated assessing the effect of the test compounds against three fungal phytopathogens (FI, FII, and FIII). The antifungal activity of the test compounds was evident from the reduction in the mycelial growth of the test fungi in poisoned plates as compared with the control plates (Figure 6). Control plates (absence of the test compounds) denoted luxuriant growth of all the three fungal pathogens on a PDA plate. In the case of FI, the application of RH-BS at the highest concentration (0.020%) hampered the growth of the pathogen by 1.68 folds. The mycelial growth of the same pathogen (FI) was inhibited by around 1.78 folds with increased concentration (0.015–0.020%) of FCHNPs. Thus, the dose-dependent inhibition of FI was observed (Figure 6FI). In the case of FII, similar kinds of observations were noted. When pathogen FII was treated with RH-BS, the growth was inhibited by 1.12 folds. Around 1.75 folds inhibition in FII mycelial growth was observed in the presence of FCHNPs at 0.015% and 0.020% concentrations (Figure 6FII). In the case of the third pathogen FIII, both RH-BS and FCHNPs inhibited mycelial growth in around 1.40 folds (at 0.015% concentration) and 1.24 folds (at 0.020% concentration), respectively (Figure 6FIII). Both the fungal pathogens (FII and FIII) were found slightly resistant since their mycelial growth was not inhibited so effectually as compared with FI. The positive control—CBD—the commercial fungicide—was found to be effective against all three fungal phytopathogens even at the lowest concentration (0.005%). The complete results are summarized in Supplementary Table S3.

#### 3.7.6 Spore Germination Method

**FIGURE 7:**
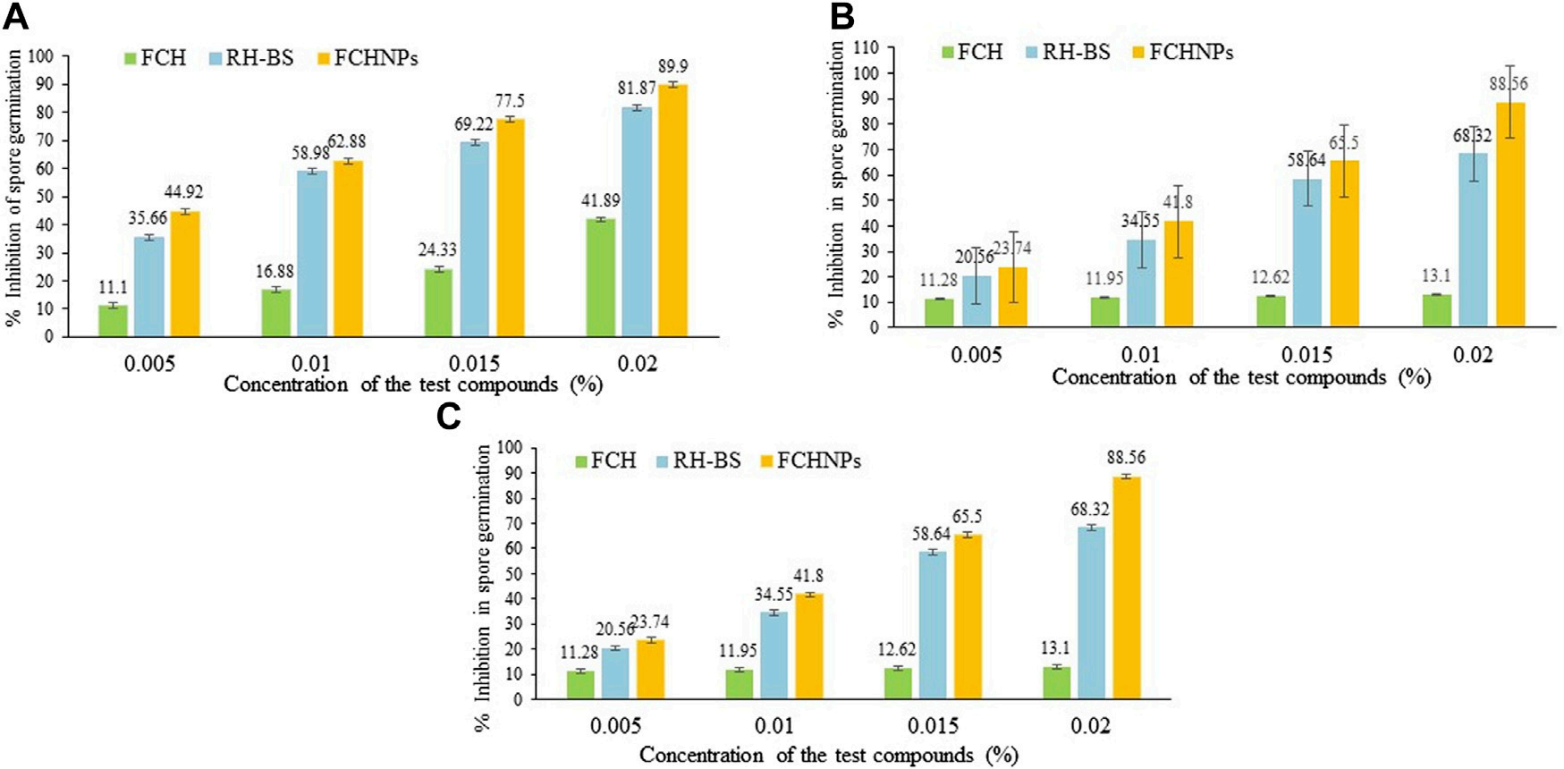
Graphical representation of inhibition in spore germination via rhamnolipid biosurfactant (RH-BS), fungal chitosan (FCH), and synthesized chitosan nanoparticles (FCHNPs) of fungal phytopathogens. **(A)** FI: *F. moniliforme* Sheldon ITCC 191; **(B)** FII: *F. moniliforme* Sheldon ITCC 4432; and **(C)** FIII: *F. graminearum* Schwabe ITCC 5334.

The effects of RH-BS, FCH, and FCHNPs on the spore germination rate of the three fungal phytopathogens are presented in Figure 7 as FI, FII, and FIII separately. Among three test compounds, the biomaterial FCHNPs significantly inhibited the spore germination in a dose-dependent manner.

The RH-BS prominently inhibited the spore germination of FI with 69.22% and 81.87% at concentrations of 0.015% and 0.020%, respectively, while FCH was found to be less effective in inhibiting the spore germination rate of the said fungus. The percentages of inhibition of 41.89% and 43% were observed when FCH was used at 0.015% and 0.020% concentrations, respectively, indicating the resistance of the fungal pathogen. It is important to note that among all the three test compounds, FCHNPs were found to be powerful antifungal agents exhibiting around 77.5% and 89.9% inhibition at concentrations of 0.0150% and 0.020%, respectively (Figure 7A).

In the case of the fungus FII, observations were similar to the FI fungal pathogen. The RH-BS inhibited the spore germination of the fungus by 56.23% and 68.32% when it was applied at a concentration of 0.015% and 0.020%, respectively, whereas FCH minimally inhibited the growth of the said pathogen by 12.62% (at 0.015% concentration) and 13.10% (at 0.020% concentration). It is noteworthy that FCHNPs are quite commanding in inhibiting the germination of spores with 65.50% and 88.56% at concentrations of 0.015% and 0.020%, respectively (Figure 7B).

In the case of the third fungal pathogen (FIII), the findings were the same as the first (FI) and the second (FII) pathogen. RH-BS maximally suppressed the spore germination of FIII by 58.64% (at 0.015% concentration) and 61.02% (at 0.020% concentration), whereas FCH inhibited the spore by 13.32% and 12.9% at concentrations of 0.015% and 0.020%, respectively. The FCHNPs were found to be again highly effective in inhibiting around 59.5% and 66.28% of the spore germination at a concentration of 0.015% and 0.020%, respectively (Figure 7C).

Overall, among the three test compounds, FCHNPs have been identified as the most potent biomaterial in inhibiting spore germination (between 59% and 90%) of all fungal phytopathogens. Followed by FCHNPs, a reasonable level of inhibition in spore germination (between 56% and 81%) was observed in all three fungi when treated with RH-BS, whereas FCH showed the lowest percentages (between 12% and 43%) in inhibition of fungal spore germination. In conclusion, all these three test biomaterials exhibited inhibition in spore germination of the fungi in a dose-dependent manner. Amongst all the three test fungi, FI was found to be affected more severely followed by FII and FIII.

## 4 Discussion

There is a constant exploration of strategies to cope with current issues related to sustainability, food security, and climate change to provide achievable solutions for agriculture. The application of innovative biomaterials for the advancement of disease management is one such approach (Sathiyabama and Parthasarathy, 2016). In recent years, antimicrobials such as RH-BS, FCH, and FCHNPs have emerged as promising candidates for controlling disease outbreaks and pathogens. Comparatively to their synthetic counterparts, BS is an effective substitute to inhibit phytopathogens (Goswami and Deka, 2021). One of the many antimicrobial compounds available is RH-BS produced by *Pseudomonas* spp. Similar to the biomaterials (FCH and FCHNPs), surface-active agents also have a dynamic role in handling a range of pathogens (Sathiyabama and Parthasarathy, 2016). All these biomaterials are considered to be valued pesticides for controlling plant pathogens in promising ways. Considering the inevitability of the current situation, we have utilized Zygomycetes fungus—*C. echinulata*, NCIM 691—to produce FCH in nutrient-rich PDB. The fungus grew luxuriously (biomass 64.2 g/L) with a high content of FCH (523.3 mg/L) on the 12th day of submerged fermentation (Figure 1). The fact that the fermentation processes dedicatedly carried out for the extraction of FCH from its biomass is comparatively costlier. Nonetheless, the anticipated features like DDA, molecular weight, and viscosity of CH for the purpose of comprehensive perspective encourage the researchers to cultivate fungal biomass on large scale. Being less explored and considering the immense biotechnological applications, *C. echinulata* is a worthwhile alternative over crustacean sources (no seasonal limitations like marine sources) for the production of FCH and FCHNPs. We have carried out the characterization of CH, CHNPs, FCH, and FCHNPs through various analytical techniques (UV-visible, DLS, zeta potential, FTIR, SEM, and NTA) and confirmed their authenticity. The size of NPs and zeta potential are crucial parameters that affect the antimicrobial properties of biomaterials (Barrera-Necha et al., 2018). The characterization study through NTA revealed a nondispersive nature and small size (144.33 ± 10.20 nm) of FCHNPs and had a zeta potential value of +45.6 mV as compared to FCH (particle size via DLS 960.6 ± 57.17 nm) and zeta potential +37 mV. The NTA also confirmed the homogeneous size of FCHNPs as compared to CHNPs (refer Figure 4). All the observations accomplished through the physicochemical characterization of both biomaterial (FCH and FCHNPs) are well comparable with the published literature (Saharan et al., 2013; Kheiri et al., 2017; Shajahan et al., 2017; Oh et al., 2019).

After completing the characterization of FCH and FCHNPs, we continued to explore their antimicrobial potential along with commercially available RH-BS against harmful phytopathogens. Even though the RH-BS has been studied extensively (with respect to producing organisms, production, optimization, physicochemical characterization), their innovative applications particularly as biocidal agent in agriculture are limited. We chose a bacterial pathogen *X. campestris*, which is a causative agent of citrus bacterial canker disease, banana Xanthomonas wilt, and black rot in cabbage, etc. (Leite and Mohan, 1990). Generally, the genera of *Xanthomonas* leads to postharvest diseases in several fruits and vegetables (Barth et al., 2009). Citrus fruits, banana, mango, and many other vegetables like tomato, cabbage, cauliflower, radish, turnip, and broccoli are usually spoiled by several *Xanthomonas* spp. The RH-BS is certainly effective in controlling the *X. campestris*. The outer membrane of *X. campestris* is composed of phospholipids, lipopolysaccharides, and lipoproteins which are covalently linked together with the peptidoglycan layer through hydrophobic interactions (Sikkema et al., 1995). The RH-BS inhibits pathogens through the damage to the cell membrane. A similar kind of observation has been reported for *Bacillus subtilis*. The mechanism of RH-BS involves an alteration in the lipid composition of the membrane due to an increase in the activity of cardiolipin (negatively charged phospholipid) and enhances membrane susceptibility of *B. subtilis* (Sotirova et al., 2012). The surface-active agent attacks pathogens and liberates their intracellular contents leading to the disruption and cell lysis (Kumar et al., 2021). The lower MIC (256 μg/ml) of RH-BS was observed against *X. campestris* as compared to FCH and FCHNPs (>1,024 μg/ml) when used individually. However, the two combination studies of RH-BS + FCH (128 μg/ml + 256 μg/ml) and RH-BS + FCHNPs (128 μg/ml + 4 μg/ml) resulted in additive (FICI is 0.75) and synergistic (FICI is 0.50) effects, respectively. Hence, our work significantly demonstrates a substantial decrease in MICs of RH-BS and FCHNPs suggesting a powerful synergistic activity against *X. campestris*.

Like bacteria, plant pathogenic fungi have acquired severe resistance against conventional agrochemicals (Gill and Garg, 2014). We conducted two antifungal assays (poisoned food assay and spore germination inhibition) for RH-BS, FCH, and FCHNPs against three fungal phytopathogens comprising *F. moniliforme* (two strains) that attack sugarcane, maize, rice, and fig and *F. graminearum* (single strain) that attack maize, wheat, and barley (Vishwakarma et al., 2013). Both techniques used by us have demonstrated a dose-dependent manner antifungal effect of RH-BS, FCH, and FCHNPs against the selected fungi (FI, FII, and FIII). The literature documents the noteworthy antifungal activity of RH-BS without harming the soil ecology unlike synthetic fungicides (Lahkar et al., 2018). Crouzet et al. (2020) reviewed the potential of RH-BS in a sustained agriculture framework suggesting the enhancement in plant immunity (local/systemic). The antimicrobial potential of RH-BS can be improved by combination with other biomaterials like FCH and FCHNPs. The NPs synthesized using biopolymer CH and BSs have attained flickered curiosity. The addition of RH-BS for the preparation of nanoconjugates facilitates a smaller and uniform size and the polydispersity index of CHNPs in productive ways. RH-BS stabilizes the CH particles and results in superior loading efficiency (Marangon et al. 2020). Generally, CH assembles actively at the surface of fungi/bacteria and permeabilizes the cell membrane through their electrostatic interactions (positive charges of CH and the negatively charged molecules at the cell surface of microbes). Consequently, the cell surface permeabilization leads to leakage of intracellular material leading to cell death (Palma-Guerrero et al., 2010; Krajewska et al., 2011). The other mechanisms involve the chelating action of CH, which binds to trace elements and thus becomes unavailable for the normal growth of microbe. The CH might penetrate through the fungal cell wall and inhibits protein and DNA synthesis (El-Naggar et al., 2022). The fungicidal action of FCH and FCHNPs is correlated with the modification of membrane permeability of the cell wall component of the pathogen (Dorocka-Bobkowska et al., 2003). The robust bonding of CH to the fungal cell wall decreases the negative surface charge of fungal membranes due to a decrease in K^+^ concentration, and this efflux disturbs the osmotic balance of the cell wall. The FCHNPs due to small particle size result in their better uptake into microbial cells and exhibit an antimicrobial effect (de Carvalho et al., 2019). The FCHNPs synthesized by us appear to bear a strong antimicrobial activity as compared with its origin material, FCH. NPs being nano in size and surface-to-charge ratio have been greatly associated with their unique functional activity (Kutawa et al., 2021). Thus, nanomaterial proves to be highly supportive in the form of nanoformulations to use as pesticides and/or fertilizers in improving crop yield. Furthermore, the nanosensors also offer applications in protecting enormous crops through the detection of agrochemicals residues (Pestovsky and Martínez-Antonio, 2017). The research conveys that the NPs act on the nucleic acids where DNA loses replication phenomena and ultimately inactivates protein expression as well as enzymes which are imperative for ATP production (Abdel-Aliem et al., 2019). The larger surface area of NPs empowers them to get adsorb firmly onto the microbial surfaces resulting in cell disruption by altering the membrane integrity. Thus, these NPs diffuse into fungal cells and inhibit the synthesis of DNA, RNA, mitochondrial function, and protein synthesis (Kutawa et al., 2021).

Most of the fungi exhibit pathogenicity and long-term survival due to their spore-forming abilities. Germination of spores is the crucial step to recruit vegetative growth and results in perilous diseases by many fungal pathogens (Ortiz et al., 2019). The unique morphology of the fungal spores is a precise target for designing and developing innovative antifungal drugs. Our study on RH-BS, FCH, and FCHNPs documents inhibition in spore germination of the selected fungi in a dose-dependent manner (0.005%, 0.010%, 0.015%, and 0.020% w/v). The inhibition of the spore germination was initiated for RH-BS and FCHNPs at 0.010% and 0.015% concentrations, respectively. Thus, RH-BS and FCHNPs pose promising applications in controlling the growth of spore-forming dreaded fungal phytopathogens. RH-BS has a repressive effect on the breeding and growth of pathogenic fungi through inhibition in spore germination (Yan et al., 2015). Recent studies have recognized the antifungal potential of RH-BS through zoospore lysis, spore germination abortion, and mycelial growth inhibition. The amphiphilic nature of BS enables them to interact with plasma membranes (Crouzet et al., 2020). The mode of action of BS is via lysis of mycelial cells resulting in their destabilization through their intercalation with the bilayers of phosphatidylcholine and phosphatidylethanolamine. Thus, the effect on membrane permeabilization is significant due to the cell lysis of the fungal pathogen. The RH-BS explicates defense responses that are induced by other elicitors like CH. Thus, these molecules cause local resistance in hemibiotrophic fungus *Leptosphaeria maculans* ultimately protecting *Brassica napus* (Monnier et al., 2020). Recently, a few members of our research group (Chopra et al., 2020) have reported promising antimicrobial activity of *P. aeruginosa* RTE4 origin RH-BS against phytopathogens *X. campestris*, *F. solani*, and *Corticium invisum.* Thus, the strain RTE4-derived RH-BS has an application as a powerful biofungicide. Through the present work, we have extended our efforts to utilize the antimicrobial potential of RH-BS with FCHNPs (from CH of fungus origin) in controlling phytopathogens. The RH-BS coupled with FCHNPs has positively improved their efficacy. The amended antimicrobial activity is related to the augmented delivery of RH-BS and FCHNPs at the cell surface of the pathogen. A similar kind of work has been reported by Marangon et al. (2020) for CHNPs (synthesized using CH of crustacean origin) in combination with RH-BS to improve antibacterial efficacy through greater electrostatic interactions and more effective disruption of the pathogen’s cell membrane. The mechanism involves the high density of polycationic NPs over the cell envelope. The stated combination study of these two biomaterials offers a promising strategy to design a biological low cytotoxic nanoformulation for the agriculture sector. When these biomaterials are used individually, they are less effective against selected pathogens; however, their synergistic activities certainly raise inquisitiveness among the research community to explore diverse innovative applications. Furthermore, in vivo assays are in the process to examine the influence of RH-BS and FCHNPs against phytopathogens.

## 5 Conclusion

In the present work, we have successfully produced the stable, valuable, and high-quality FCH from the fungal mycelia of *C. echinulata* NCIM 691. Furthermore, FCHNPs were synthesized magnificently using *C. echinulata*-originated FCH through the ionic gelation method. Matricular physicochemical characterization of FCH and FCHNPs assured their authenticity. The improvised antibacterial activity of RH-BS and FCHNPs was observed in combination against *X. campestris.* Promising antimicrobial activity of RH-BS with FCHNPs against selected phytopathogens encouraged us to recommend these types of novel bioformulation for the future. Plenty of monitoring protocols is obligatory to utilize nanoagrochemicals as active substance, coformulants, with or without other adjuvants. The difficulty in tracking the role/mechanism of these nanocarriers in food and the environment gratifies the use of biologically originated NPs. It is important to highlight that not much work has been reported so far in deliberating the biocontrol potential of RH-BS with FCH and FCHNPs for their synergistic and additive activity against phytopathogens associated with staple (rice, wheat, maize, and barley) and commercially important crops (citrus fruits, mango, banana, date palm fruits, cruciferous vegetables, and turfgrass, etc.). Our work demonstrating the synergistic activity of RH-BS with FCHNPs is crucial in facilitating eco-friendly formulations against harmful phytopathogens.

## Data Availability

The raw data supporting the conclusion of this article will be made available by the authors, without undue reservation.

## Footnotes

1
https://www.prnewswire.com/news-releases/global-agrochemicals-market-2021-to-2027.

## Abbreviations

BSs, biosurfactants; CH, chitosan; CBD, carbendazim; CHNPs, chitosan nanoparticles; CFU, colony-forming unit; DDA, degree of deacetylation; FCH, fungal chitosan; FCHNPs, fungal chitosan nanoparticles; LB, Luria–Bertani broth; MIC, minimum inhibitory concentration; MGYP, malt extract glucose yeast extract peptone; MHB/MHA, Mueller–Hinton broth/agar; NPs, nanoparticles; PBS, phosphate buffer saline; RH, rhamnolipids; SEM, scanning electron microscope; TPP, sodium tripolyphosphate.
