# Supplementary figures and images for: Synergistic Activity of Rhamnolipid Biosurfactant and Nanoparticles Synthesized Using Fungal Origin Chitosan Against Phytopathogens
Source: Front Bioeng Biotechnol. 2022 Aug 9;10:917105. doi: 10.3389/fbioe.2022.917105 (PMC9396382; doi:10.3389/fbioe.2022.917105)

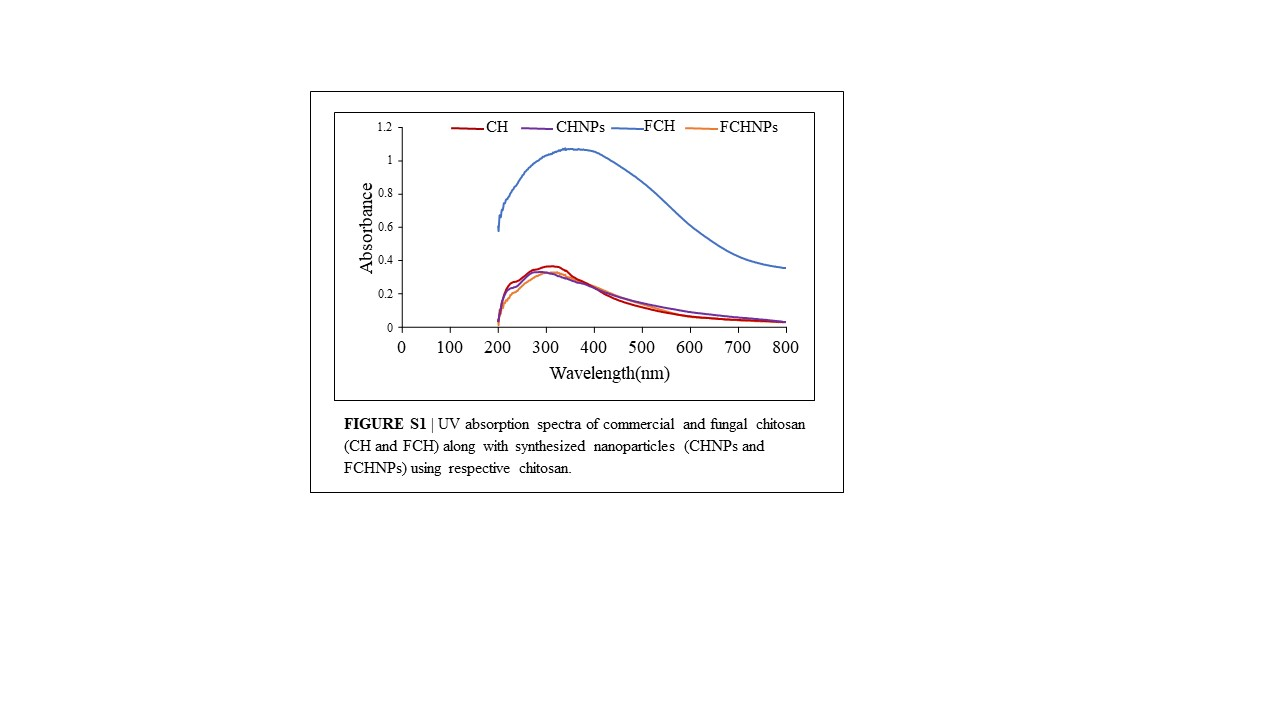

Supplement: Supplementary file 1 [file Image1.TIF]
