# Supplementary material for: Synergistic Activity of Rhamnolipid Biosurfactant and Nanoparticles Synthesized Using Fungal Origin Chitosan Against Phytopathogens
Source: Front Bioeng Biotechnol. 2022 Aug 9;10:917105. doi: 10.3389/fbioe.2022.917105 (PMC9396382; doi:10.3389/fbioe.2022.917105)
Supplement: Supplementary file 2 [file DataSheet1.pdf]

TABLE 1 | The size and zeta potential analysis of commercial and fungal chitosan and respective synthesized nanoparticles.

Chitosan source	Hydrodynamic diameter (nm)(Dynamic Light Scattering)	Zeta potential (+) (mV)	Nanoparticle Size Analysis (nm)
CH	918.2±17.69	23.6	NR
CHNPs	354.6 ±25.61	24.5	256.33±18.80
FCH	960.6±57.17	37	NR
FCHNPs	229.5±47.44	45.6	144.33±10.20

CH: Commercially available chitosan, CHNPs: Synthesized nanoparticles from commercially available chitosan, FCH: Fungal chitosan, FCHNPs: Synthesized nanoparticles from the chitosan extracted from the fungus *C. echinulata* NCIM 691, NR: Not required.

TABLE 2 | A fractional inhibitory concentration index of the test compounds against *X. campestris* NCIM 5028.

Combinations of the test compound	MIC	FICI	Activity
1. RH-BS + FCH	Individual- RH-BS: 256 µg/ml; FCH: >1024 µg/ml<br>Combination- RH-BS: 128 µg/ml & FCH: 256 µg/ml	0.75	Additive
2. RH-BS + FCHNPs	Individual- RH-BS: 256 µg/ml; FCHNPs: >1024 µg/ml<br>Combination- RH-BS: 128 µg/ml & FCHNPs: 4 µg/ml	0.503	Synergistic

RH-BS: Rhamnolipid-Biosurfactant, FCH: Fungal chitosan, FCHNPs: Synthesized nanoparticles from the chitosan extracted from the fungus *C. echinulata* NCIM 691.

TABLE 3 | Inhibition percentage in fungal growth on potato dextrose medium impregnated with the test compounds.

Name of the fungal pathogen	Control: No test compound (0%)	Inhibition percentage in fungal growth on potato dextrose medium impregnated with the test compound at different concentrations (%)															
		Concentration: 0.005%				Concentration: 0.010%				Concentration: 0.015%				Concentration: 0.020%			
		RH-BS	FCH	FCH NP	CBD	RH-BS	FCH	FCH NP	CBD	RH-BS	FCH	FCH NPs	CBD	RH-BS	FCH	FCH NP	CBD
FI: *F. moniliforme* ITCC 191	NI	NI	NI	NI	100	23.9± 1.10	26.2± 3.72	26.4± 1.14	100	45.1± 0.82	NI	54.6± 1.10	ND	71.1± 3.84	32.6±0.47	92.6±0.53	ND
FII: *F. moniliforme* ITCC 4432	NI	NI	NI	NI	100	NI	NI	NI	100	53.7± 1.22	NI	53.3± 1.06	ND	81.9± 0.53	NI	90.8±1.14	ND
FIII: *F. graminearum* ITCC 5334	NI	NI	NI	NI	100	NI	NI	NI	100	49.9± 0.45	NI	62.6± 0.094	ND	60.2± 1.18	NI	77.1±0.82	ND

RH-BS: Rhamnolipid-Biosurfactant; FCH: Fungal Chitosan; FCHNPs: Synthesized nanoparticles from the chitosan extracted from the fungus *C. echinulata* NCIM 691; CBD: Carbendazim - commercial pesticide; NI: No inhibition; ND: Not done. Each value is the mean of 3 replicates from each experiment according to concentration change. Mean ± SE is done which does not have a significant difference at $p<0.05$.
